# Dietary intake and main sources of plant lignans in five European countries

**DOI:** 10.3402/fnr.v57i0.19805

**Published:** 2013-06-11

**Authors:** Inge Tetens, Aida Turrini, Heli Tapanainen, Tue Christensen, Johanna W. Lampe, Sisse Fagt, Niclas Håkansson, Annamari Lundquist, Jesper Hallund, Liisa M. Valsta, The Phytohealth WP1 working group

**Affiliations:** 1 Division of Nutrition, National Food Institute, Technical University of Denmark, Søborg, DK, Denmark; 2 National Institute for Research on Food and Nutrition, Rome, IT, Italy; 3 National Institute for Health and Welfare, Helsinki, FI, Finland; 4 Fred Hutchinson Cancer Research Centre, Cancer Prevention Program, Seattle, WA, USA; 5 Institute of Environmental Medicine, Karolinska Institute, Stockholm, SE, Sweden; 6 European Food Safety Authority, Parma, IT, Italy

**Keywords:** lignan intake, secoisolariciresinol, matairesinol, lariciresinol, pinoresinol

## Abstract

**Background:**

Dietary intakes of plant lignans have been hypothesized to be inversely associated with the risk of developing cardiovascular disease and cancer. Earlier studies were based on a Finnish lignan database (Fineli^®^) with two lignan precursors, secoisolariciresinol (SECO) and matairesinol (MAT). More recently, a Dutch database, including SECO and MAT and the newly recognized lignan precursors lariciresinol (LARI) and pinoresinol (PINO), was compiled. The objective was to re-estimate and re-evaluate plant lignan intakes and to identify the main sources of plant lignans in five European countries using the Finnish and Dutch lignan databases, respectively.

**Methods:**

Forty-two food groups known to contribute to the total lignan intake were selected and attributed a value for SECO and MAT from the Finnish lignan database (Fineli^®^) or for SECO, MAT, LARI, and PINO from the Dutch database. Total intake of lignans was estimated from food consumption data for adult men and women (19–79 years) from Denmark, Finland, Italy, Sweden, United Kingdom, and the contribution of aggregated food groups calculated using the Dutch lignin database.

**Results:**

Mean dietary lignan intakes estimated using the Dutch database ranged from 1 to 2 mg/day, which was approximately four-fold higher than the intakes estimated from the Fineli^®^ database. When LARI and PINO were included in the estimation of the total lignan intakes, cereals, grain products, vegetables, fruit and berries were the most important dietary sources of lignans.

**Conclusion:**

Total lignin intake was approximately four-fold higher in the Dutch lignin database, which includes the lignin precursors LARI and PINO, compared to estimates based on the Finnish database based only on SECO and MAT. The main sources of lignans according to the Dutch database in the five countries studied were cereals and grain products, vegetables, fruit, berries, and beverages.

Plant lignans are plant-derived diphenolic compounds that belong to the group of phytoestrogens that are structurally similar to 17-estradiol. After ingestion, plant lignans are metabolized to the enterolignans enterodiol (END) and enterolactone (ENL) by colonic bacteria before they are absorbed (1, 2). END and ENL are detected in plasma within 8–10 h after intake of plant lignans (3), and their half-lives in plasma are approximately 5 and 13 h, respectively (4, 5). However, a substantial inter-individual variation has been detected in plasma concentrations and urinary excretion of enterolignans, partly due to the complex interaction between colonic environment and external and internal factors (6) which moreover, seems to be more dependent on the dietary lignan source than the absolute lignan intake (7). Reliable methods of exposure measurement are crucial for understanding the possible health benefits of plant lignans and the first step in this process is to establish comprehensive dietary databases to estimate plant lignan exposure in population-based studies (6).

Observational studies have examined the association between habitual intake of plant lignans – estimated from the intake of selected food items and their content of two major precursors of enterolignans secoisolariciresinol (SECO) and matairesinol (MAT) – and risk of developing lifestyle-related diseases, such as cardiovascular disease (8, 9), breast cancer (10), and prostate cancer (11). The metabolite responsible is ENL showing an inverse association with postmenopausal breast cancer risk (12) and mortality risk due to coronary heart disease, cardiovascular disease (13), and breast cancer (14) at high ENL serum levels. Furthermore, animal experiments on rats (15) and studies *in vitro* showed a breast cancer protective effect of END and ENL that is discussed to be imputable to their higher biological activity (16). Dietary lignan intake was also found to decrease the risk of adenocarcinoma of the esophagus and gastroesophageal junction on a case-control study (17).

As progress has been made in this area, a food database of Dutch plant foods was published (18) with data on the content of SECO and MAT, as well as two more recently identified precursors of mammalian lignans, lariciresinol (LARI) and pinoresinol (PINO) (19). Estimated dietary intakes of lignans in the Dutch diet based on the new food database of Dutch plant foods (20) suggest that plant lignan intakes are much higher than first reported (21–24) and that LARI and PINO contribute approximately 75% to the estimated intake of plant lignans in the Dutch diet with the primary food group sources of lignans being beverages, vegetables, nuts and seeds, bread and fruits (20). A few oilseeds, such as flaxseed and sesame seeds have a high content of plant lignans (18), but the intake of such foods is only used by a small proportion of the population and commonly, the amounts consumed are low (20).

In addition to the improved understanding of the importance of total lignan intakes, it has become clear that the contribution of selected food groups to the total plant lignan intake may be different than originally expected due to the contribution from LARI and PINO. Therefore, it is necessary to re-estimate and re-evaluate the total intake of plant lignans and contribution from different food groups to the total intakes in other European countries.

The main objective of this study is to estimate the total intake of plant lignans and identify the main food sources of plant lignans in different European countries by using a Finnish lignan database (Fineli^®^) which includes two enterolignan precursors MAT and SECO and a Dutch lignan database which includes four enterolignan precursors SECO, MAT, LARI, and PINO.

## Methods

The lignan intakes were calculated from 42 food groups that included plant foods and beverages known to be sources of lignans among European men and women. Each of the 42 food groups were given a lignan value for the content of the mammalian lignan precursors MAT, SECO, PINO, and LARI based on the lignan database of Dutch plant foods (18) or on only MAT and SECO based on the Fineli^®^ database from Finland (24) as described in Appendix A. Both databases provide the Linnean binomial nomenclature for plants. The new lignan values for food groups were either weighted values or arithmetic means based on the food content of plant lignans available from commonly consumed food items best representing that food group. For example, in the case of Fineli^®^-based values, the lignan content of whole grain rye flour contributed most to the food group ‘rye’, whereas the value for cabbages was the arithmetic mean of all available lignan values for different types of cabbages. In the case of food group values based on the Dutch database, the food group values were based on a single analyzed food item (e.g. value of strawberry for the group ‘berries’) or arithmetic means drawn from the analyzed values available in the database (e.g. mean of tofu and soy milk for the group ‘soy products’).

Food consumption data for men and women were available for Denmark, Finland, Italy, Sweden, and the United Kingdom. The data included individual data from national dietary surveys (DK, FI, IT, UK) and from cohort studies in Sweden. An overview of the studies is presented in Table 1. All analyses were performed by using SPSS statistical software package (version 12, Chicago). The non-parametric Kruskal-Wallis ranked test was used to test the differences in the distribution of the total lignan intakes between Denmark, Finland, and Italy. Lignan intakes and source estimates were calculated by Microsoft Office Excel (2003).

**Table 1:** Description of the food consumption data

Country	Year	Dietary data level	*N*, age	Methodology used	Reference
Denmark	2000–2002	National dietary survey, data at individua. level	F: 1,307; M: 1,156 25–64 years	7-day pre-coded food record	(25)
Finland	2002	National dietary survey (FINDIET), data at individual level	F: 1,095; M: 912 25–64 years	48-h dietary recall	(26)
Italy	1994–1996	National dietary survey, data at individual level	F: 682; M: 586 25–64 years	7-day mixed survey technique	(27)
Sweden	1987–1990	Cohort studies (Swedish Mammography Cohort (SMC) & Cohort of Swedish Men (COSM), data at group level	F: 37,854; M: 45,906 45–79 years	96-item food frequency questionnaire (FFQ)	(28)
United Kingdom	2000–2001	National dietary survey, data at individual level	F: 958; M: 766 19–64 years	7-day dietary record	(29)

F = females; M = males.

## Results

The mean lignan intake estimated using the Dutch lignan database with the four lignan precursors SECO, MAT, LARI, and PINO was lowest among Finnish women (1,036 µg/day) and highest among Swedish men (1,947 µg/day) (Table 2). It is noticeable that the SECO and MAT figures are systematically smaller when calculated using the Dutch data set compared with the Finnish database. SECO contributed between 11 and 22% to the lignan intake, MAT between 1 and 3%, LARI between 41 and 45% and PINO between 32 and 44%. The total lignan intake was approximately four times higher when the estimates were calculated using the Dutch lignan database compared to the results based on the Finnish lignan database (Fineli^®^ database). According to the latter estimates, the mean lignan intake was lowest among Italian women (272 µg/day) and highest among Danish women (439 µg/day) and SECO contributed between 81 and 94% to the lignan intake and MAT between 6 and 19%.

**Table 2:** Lignan intakes (g/day) from five European countries calculated using the Dutch and the Finnish (Fineli^®^) lignan database (mean values)

	Dutch lignan database					Finnish (Fineli^®^) lignan database		
		
Country	Estimated lignan intake (g/day)					Estimated lignan intake (g/day)		
	SECO	MAT	LARI	PINO	Total	SECO	MAT	Total
Denmark								
All (*n =* 2,463)	314	41	630	473	1,459	375	57	432
Female (*n =* 1,307 )	314	43	641	486	1,484	380	59	439
Male (*n*=1,156)	315	38	618	459	1,430	370	54	424
								
Finland								
All (*n*=2,007)	188	23	469	401	1,081	245	40	285
Female (*n*=1,095)	176	21	455	384	1,036	245	34	279
Male (*n*=912)	202	26	486	422	1136	246	48	293
								
Italy								
All (*n*=1,268)	143	11	500	467	1,120	290	19	309
Female (*n*=682)	125	9	477	452	1,062	257	16	272
Male (*n*=586)	165	14	527	484	1,188	329	23	351
Sweden								
All (*n*=83,760)	224	37	735	777	1,773	318	60	377
Female (*n*=37,854)	203	28	657	675	1,563	300	39	339
Male (*n*=45,906)	242	45	799	861	1,947	332	77	409
								
United Kingdom								
All (*n*=1,724)	205	19	535	480	1,239	267	17	285
Female (*n*=891)	197	19	507	450	1,173	265	17	282
Male (*n*=833)	214	19	570	518	1,321	270	18	288

**Fig. 1:**
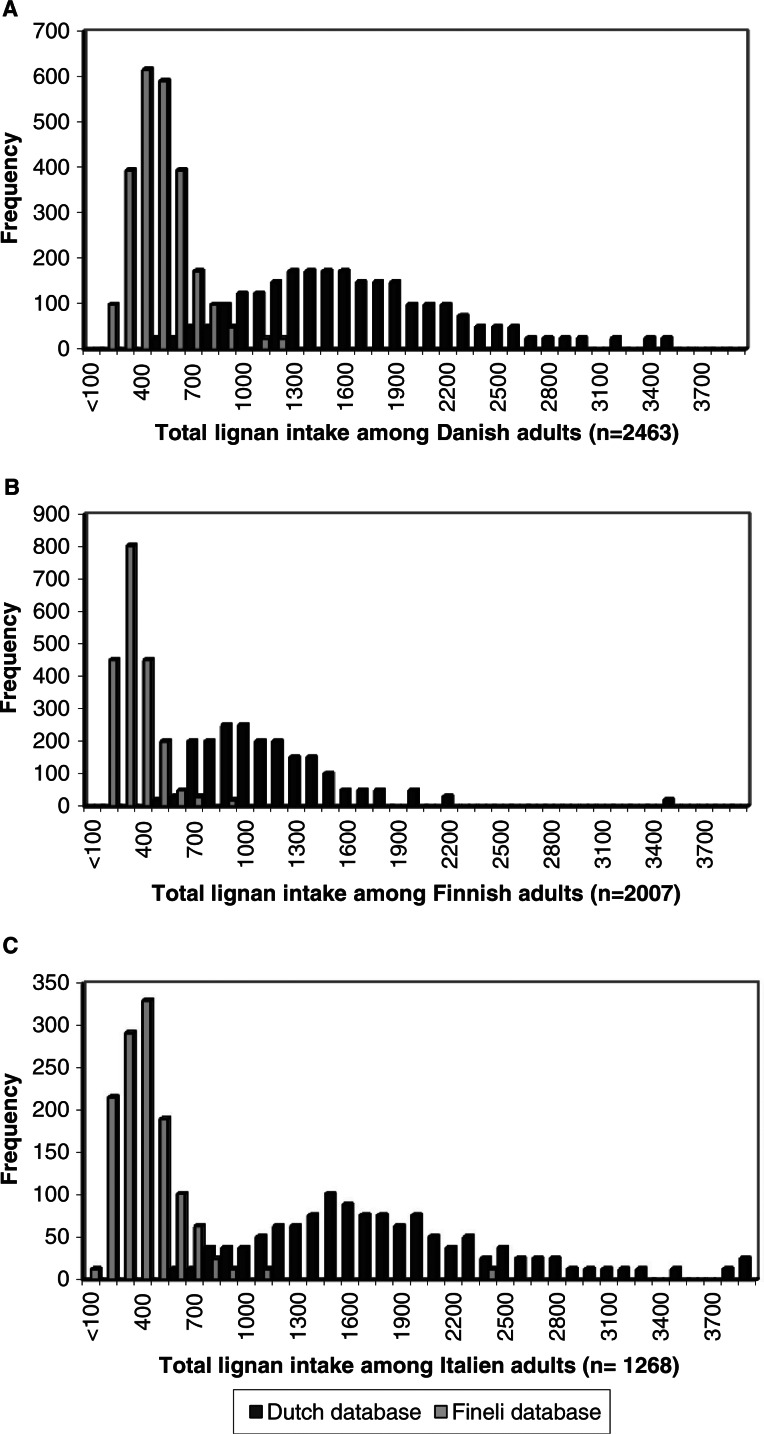
Distribution of estimated lignan intake (µg/day) among adults in Denmark (A), Finland (B), and Italy (C) calculated using the Dutch and the Finnish (Fineli^®^) lignan database.

The frequency distribution of the estimated total lignan intake among Danish, Finnish, and Italian adults using the Dutch and Finnish (Fineli^®^) lignan databases is shown in Fig. 1. The mean lignan intake between countries estimated using the Dutch lignan database of plant foods ranged from 404 µg/day among Finnish adults to 569 µg/day among Italian adults and was slightly skewed toward higher values. The mean lignan intake estimated using the Finnish (Fineli^®^) lignan database within one country ranged from 91 to 2,335 µg/day among Italian adults and was strongly skewed toward higher values. Significant differences were found between the three countries in the total lignan intakes estimated using both the Dutch database (*P*<0.001) and the Finnish (Fineli^®^) lignan databases (*P*<0.001) (data not shown).

The main food groups and food items contributing to the lignan intake estimated using the Dutch lignan database are shown in Table 3. Only food groups and food items, that contributed more than 5% to total lignan intake, have been included in the table. The most important food groups were ‘cereals and grain products’, ‘vegetables’, and ‘fruit and berries’. Cereals and grain products contributed 15–43% of total lignan intake, vegetables16–30%, and fruit and berries 15–46%.

**Table 3:** Contribution of aggregated food groups and individual foods to the total lignan intake by gender in five European countries calculated using the Dutch lignan database^1^(%)

	Denmark		Finland		Italy		Sweden		United Kingdom	
					
Food groups	Men (%)	Women (%)	Men (%)	Women (%)	Men (%)	Women (%)	Men (%)	Women (%)	Men (%)	Women (%)
Cereals and grain products	30	27	36	27	17	17	43	26	17	15
*Rye*	*17*	*21*	*24*	*17*	*0*	*0*	*0*	*0*	*0*	*0*
*Wheat*	*7*	*4*	*7*	*6*	*11*	*9*	*24*	*12*	*8*	*6*
*Other grains*	*2*	*2*	*1*	*1*	*1*	*2*	*5*	*4*	*8*	*8*
*Crisp breads*	*0*	*0*	*2*	*2*	*3*	*5*	*14*	*10*	*0*	*0*
										
Vegetables	19	20	16	20	26	28	18	30	23	25
*Cabbages*	*5*	*5*	*4*	*6*	*5*	*5*	*11*	*19*	*16*	*17*
*Fruit vegetables*	*5*	*6*	*6*	*7*	*15*	*16*	*2*	*3*	*3*	*3*
*Onion-family vegetables*	*5*	*5*	*3*	*3*	*2*	*2*	*2*	*2*	*0*	*0*
										
Fruit and berries	18	25	22	31	42	46	15	23	15	20
*Citrus fruit*	*1*	*2*	*4*	*6*	*4*	*4*	*2*	*3*	*1*	*1*
*Malaceous and prunus fruit*	*1*	*2*	*5*	*8*	*36*	*38*	*7*	*10*	*6*	*7*
*Other fruit*	*14*	*19*	*4*	*5*	*1*	*1*	*2*	*4*	*5*	*7*
*Berries*	*1*	*1*	*5*	*8*	*1*	*2*	*4*	*5*	*2*	*4*
Beverages	21	21	17	17	4	5	19	18	30	32
*Coffee*	*15*	*12*	*12*	*10*	*3*	*3*	*9*	*9*	*11*	*10*
*Tea*	*5*	*9*	*5*	*8*	*1*	*2*	*10*	*9*	*19*	*22*
										
Alcohol beverages	11	5	5	2	9	4	5	2	10	4
*Beers*	*6*	*1*	*4*	*1*	*1*	*0*	*4*	*1*	*8*	*1*
*Wine*	*5*	*4*	*1*	*1*	*8*	*3*	*1*	*1*	*2*	*3*

1 Only food groups and food items, which contributed to more than 5% of the total lignan intake at least in one of the countries, are included.

The Dutch food database includes the lignan precursors; secoisolariciresinol (SECO), matairesinol (MAT), lariciresinol (LARI), and pinoresinol (PINO).

Beverages were an additional major source of lignans in all countries. The importance of selected food items varied across countries. Rye was the most important contributor to the lignan intake in the Scandinavian countries Denmark and Finland, whereas wheat and other grains were more important in Sweden, Italy, and United Kingdom. Cabbages were important contributors to lignan intake in Sweden and United Kingdom, whereas the group ‘fruit vegetables’ (e.g. sweet pepper, tomatoes) contributed most to the lignan intake from vegetables in Italy. Malaceous and prunus species fruits (e.g. apricot, peach, pear, and nectarines) were the most important contributors to lignan intake in Italy but were less important contributors in other counties. Tea was the most important contributor to lignan intake in the United Kingdom and in combination with coffee, was a major source to lignan intake in all countries. Among men in Denmark, Italy, and United Kingdom, alcoholic beverages, especially beer and wine contribute to about 10% of the total lignan intake (Table 3).

## Discussion

The estimated mean lignan intake was approximately fourfold higher when calculations were based on the Dutch lignan database of plant foods including four lignan precursors compared with the calculations using the Finnish lignan database (Fineli^®^) that includes two lignan precursors SECO and MAT. The additional contribution to the mean lignan intake from the two additional precursors LARI and PINO was 41–45% and 32–44%, respectively. These results are in accordance with data from other investigators concluding LARI and PINO to present >70% of the total lignan intake (30). Dietary lignan intake further was more strongly associated with plasma enterolignan concentrations when taking all four mammalian lignans into account (31).

Estimations of lignan intake based on the Dutch lignan database showed that the major sources of lignans in Europe are from the food groups: ‘cereals and grain products’, ‘vegetables’, ‘fruit and berries’, and ‘beverages’.

In this study, we introduced a relatively simple approach to estimate total lignan intake when food intake data are available. In our approach, average lignan values were applied to food groups that are common in food databases. We used food groups that are known to contribute considerably to the total lignan intake and aggregated them into 42 food groups. The total amount of lignan intake was calculated based on the aggregated amounts of food consumed and the average weighted lignan content of that food group.

In this study, the estimated total lignan intakes based on the Dutch lignan database including four lignan precursors were of similar range as an earlier estimate of the lignan intake of 979 µg/day among Dutch men and women aged 19–97 years (20) and very recent estimates among Finnish men (7) and Italian men and women (32). Compared to the total lignan intakes among Dutch men and women, the total lignan intakes were higher in Denmark and Sweden and within similar range in Finland, Italy, and United Kingdom. The high lignan intakes in Denmark and Sweden were mainly due to a higher consumption of rye and wheat products, respectively. In all Scandinavian countries, cereals and grain products are important contributors to lignan intake whereas fruits and berries are main contributors in Italy and beverages (tea, coffee, and beer) are main contributors in the United Kingdom.

The inclusion of the precursors LARI and PINO in the estimated total lignan intake has shown that more food groups contribute to the total lignan intakes than earlier expected. Vegetables, fruit, and berries are important contributors to the total lignan intakes because they have a relatively high content of LARI and PINO (18). When using four lignan precursors, LARI, and PINO were the main contributors to the lignan intake in all five countries. This has been confirmed also in more recent studies (7, 32).

In earlier studies, where the lignan intakes was estimated based on MAT and SECO, the major contributor to the lignan intakes was grain products, whereas tea, coffee, nuts, seeds, and selected fruits and vegetables only contributed to a smaller proportion of the intake (21, 22, 33). The systematically lower values for the SECO + MAT intake values estimated using the Dutch database compared with the values estimated from the Finnish database can be explained mainly by the different analytical methods (18, 24).

Some issues need to be discussed in order to fully appreciate the results. First, the 42 food groups were selected because they are important contributors to lignan intake based on former knowledge (24). For each of the 42 food groups, a lignan value was chosen to represent the lignan content of all foods from that food group. The decisions on which these plant lignan values were chosen are provided in Appendix A. The lignan values were calculated from a mean of all foods from a particular food group or from a weighted average. An average was weighted according to the importance of foods consumed from a particular food group and taken into account that certain single foods such as sesame seeds and flaxseeds have a high lignan content. It should be noted that the approach used in this study results in a relatively narrow range of lignan intake. Furthermore, both food databases used in this study have been developed from analyses of locally representative foods in the Netherlands and Finland, respectively. Possible differences in the lignan content of country-specific foods due to differences in types of foods available, preparation of foods, available brands are not taken into account in this study. Finally, the fact that the food consumption data were collected using three different dietary assessment methods, i.e. 7-day food records in Denmark, Italy, and the United Kingdom, a 48-h dietary recall in Finland, and a food frequency questionnaire (FFQ) in Sweden, the results are not directly comparable. This may have led to a larger variation and differences in the estimated lignan intakes across countries. However, the results also reflect different dietary patterns and different food intakes.

In conclusion, we have shown that the total lignan intake was approximately fourfold higher after inclusion of the two new mammalian lignan precursors, LARI and PINO, when compared to estimates based on only SECO and MAT. Furthermore, we have shown that LARI and PINO contributed the majority of the lignan intakes in all five countries. When LARI and PINO were included in the estimation of the total lignan intakes, the major sources of lignans were cereals and grain products, vegetables, fruit, berries, and beverages.

## Appendix

**Appendix A:** Content of lignan and lignan precursors (SECO, MAT, LARI, PINO) in foods and notes on which the lignan values were chosen

	Finnish (Fineli^®^) database				Dutch database					
			
	Total lignan ug/100g	SECO ug/100g	MAT ug/100g	Notes	Total lignan ug/100g	LARI ug/100g	PINO ug/100g	SECO ug/100g	MAT ug/100g	Notes
**Cereal and grain products**										
Rye	95	40	55	Weighted mean (whole grain rye flour)	458	175	246	23	20	Ryebread x 1.43
Wheat	25	20	5	Weighted mean (wheat flour)	99	60	29	12	0	Wheatbread x 1.43
Oats and barley	18	13	5	Mean (rolled oats)	107	60	29	13	5	Estimate based on FINMAT&SECO and the proportions of new precursors
Rice	27.4	26.4	1	Mean of foods in the group of rice/rice containing foods	23.5	17.5	3.5	1.5	1	Mean of white rice and whole grain rice
Pasta and macaroni	14.5	11.6	2.9	Mean of all pastas	16	7	5	4	0	Cooked pasta
Other grains	16	15	1	Weighted mean (millet, corn, buckwheat)	485	157	313	15	0	Mean of 3 müslies
Crispbreads (as eaten)	52.4	27.4	25	Mean of foods in the food group, MAT-value weighted by rye crisp bread	412	156	221	21	18	Rye flour x 0.9
Biscuits (as eaten)	7.2	6.8	0.4	Mean of all foods in the group	18	9	4	5	0	White flour (taking into account the moisture)
**Potatoes**										
Potato	3.2	2.0	1.2	Weighted mean (potato)	16	10	0	4	2	Potatoes (seco and matai estimates based on Fineli and proportions of new precursors)
Potato products	3.2	2.0	1.2	Weighted mean (french fries)	16	10	0	4	2	Potatoes
**Vegetables**										
Root vegetables and tubers	17.5	16.6	0.9	Mean of root vegetables and tubers (excluding dried carrots)	88	31.5	9.5	47	0	Mean of carrot and red beet
Leafy vegetables	30.5	30	0.5	Weighted mean (lettuce)	57	35	12.5	9.5	0	Mean of spinach, chicory, endive, lettuce, Iceberg lettuce
Cabbages	30.5	30.3	0.2	Mean of the group	600	255	335	8	2	Weighted mean (most common cabbages)
Fruit vegetables	5.5	5.49	0.01	Weighted mean (tomato and cucumber)	132	103	19	10	0	Mean of sweet pepper, zucchini, cucumber and tomato
Onion-family vegetables	23.8	20	3.8	Mean, Seco-value weighted by onion	287	153	100	34	0	Mean of garlic, leek and onion
Canned vegetables	20	20.0	0.02	Mean of processed vegetables (excluding pickled pumpkin)	104	58.3	40.0	5.3	0	Mean of corn and pea
Edible fungi	6.0	2.4	3.7	Mean of the food group	0	0	0	0	0	Mushroom
**Pulses and nuts**										
Pulses	4.5	4.4	0.1	Weighted mean (pea)	93	67	14	12	0	Estimate according to beans
Nuts and seeds	300	299	0.9	Weighted mean (almond, nuts)	287	8.2	25.3	253	0.5	Mean (nuts)
Soy products	30.5	30	0.5	Estimate according to major soy containing foods (soy flour, soy beans)	88.9	33.8	45.5	9.6	0	Mean of tofu and soy milk
**Fruits**										
Citrus fruit	14.6	14.6	0.02	Mean of all citrus fruit	112	71	33	6	1.5	Mean of grape fruit, mandarine and orange
Malaceous and prunus species fruits	70.3	70	0.3	Mean of all malaceous and prunus species fruits	251	78	157	15.5	0	Mean of apricot, peach, pear, nectarine, prunes and apple
Other fruits	91.8	87.3	4.4	Mean of all other fruit	192	76.5	50	56	9.5	Mean of raisins, cherries, kiwi, olives, melons, grapes, pineapple and banana
Canned fruits	55.6	51.2	4.4	Mean of canned fruit	20	3	5	7	5	Canned pineapple
Berries	188	186	2.3	Mean of all berries	334	117	212	5	0	Strawberry
Juices	17	15	2	Weighted mean (orange juice)	18.1	5.4	4.1	6.7	1.9	Mean of grape, tomato, orange and grape fruit juices
Juice drinks	10.3	10	0.3	Weighted mean (berry juices)	35.5	3	1.5	30	1	Estimated according to Fineli berry juices and Milder et al. Berry values
**Fats**										
Oils	0.7	0.6	0.1	Mean of oils	124	2.5	122	0	0	Mean of olive, soy and sunflower oils
Margarine and fat spread	0.01	0.01	0	Mean of margarines	39	7	0	32	0	Margarine
**Beverages**										
Coffee	10	10	0	Estimate according to Milder et al. and Mazur et al.	25	11.1	0.95	12.7	0.35	Mean of three analyzed coffees
Tea	6	5	1	Calculated according to Mazur et al.	58.4	24.8	23.2	9.00	1.6	Mean of three black and one green teas
Soft drinks	1.3	1.3	0		0	0	0	0	0	Cola drink
**Alcoholic beverages**										
Beers	1	1	0	Estimate according to Milder et al.	25.5	7.6	17.4	0.5	0	Mean of three lager bears
Wines	62.4	56.9	5.5	Mean of all foods in the group	55.7	10.4	6.8	33.3	5.3	Mean of three red and three white wines
**Sugar and confectionery**										
Sugar and syrups	0	0	0		0	0	0	0	0	
Other sugar products	5.3	5	0.3	Estimate according to nutspread and licoridgesauce	0	0	0	0	0	
Non-chocolate confectionery	19.9	19.0	0.8	Mean (excluding halva)	0	0	0	0	0	
Chocolate	10.1	10	0.1	Weighted mean (milk chocolate and mean chocolate)	43	20	23	0	0	Dark chocolate
**Spices**										
Dried herbs	297	295	1.5	Mean of all in the food group	0	0	0	0	0	
Dried spices	0	0	0		0	0	0	0	0	
Condiments	27.9	27.7	0.3	Mean of all in the food group	0	0	0	0	0	
**Manufactured foods**										
Snacks	7.6	6.2	1.4	Mean of all in the food group	0	0	0	0	0	
Chocolate powder	32.9	32.9	0	From the Fineli database	60	26	26	8	0	Cocoa powder
